# EHMTI-0306. Contact heat-evoked potentials (CHEPS) in healthy subjects and patients with episodic or chronic migraine

**DOI:** 10.1186/1129-2377-15-S1-D54

**Published:** 2014-09-18

**Authors:** SL Sava, T Sasso D'Elia, R Baschi, E Vecchio, V de Pasqua, J Schoenen, D Magis

**Affiliations:** 1 Department of Neurology, University of Liège, Liege, Belgium

## Introduction

Habituation and 1st block amplitude of sensory evoked potentials are commonly reduced in episodic migraine between attacks, whereas in chronic migraine 1st block amplitude is increased and habituation tends to normalize like during an attack. The aim of our study was to compare Contact Heat-Evoked Potentials (CHEPs) in healthy subjects (HS), episodic migraine without aura (EM) and chronic migraine (CM).

## Methods

Ninety subjects participated in the study: 53 HS, 31 EM and 6 CM. CHEPs were obtained using 53°C stimuli on the forehead and the wrist. Twenty responses were averaged and partitioned in 5 blocks of 4 responses. We measured P1, N2 and P2 latencies and P1-N2 and N2-P2 amplitudes and habituation. Pain was rated using a visual analog scale (VAS). Data were analyzed with ANOVA and post-hoc Scheffe’s test.

## Results

There was no significant difference in CHEPs latency, amplitude or habituation between HS and EM either in the forehead or at the wrist. By contrast, in CM patients P1-N2 amplitude after forehead stimulation was increased (p=0.04) and habituation more pronounced (p=0.04). Both at forehead and wrist VAS pain ratings were significantly higher in CM than in HS (p=0.02) or EM (p=0.01).

## Conclusion

We found no difference between HS and interictal EM for thermonociceptive evoked potentials. Contrasting with these findings, CM patients display increased CHEPs amplitude, CHEPs habituation and pain perception. Such a pattern is similar to that found in these patients with visual evoked responses and may be due to central sensitisation and increased thalamo-cortical drive.

No conflict of interest.

